# Disruption of daily rhythms in gene expression: The importance of being synchronised

**DOI:** 10.1002/bies.201400043

**Published:** 2014-05-16

**Authors:** Alun T L Hughes, Hugh D Piggins

**Affiliations:** Faculty of Life Sciences, University of ManchesterManchester, UK

**Keywords:** circadian rhythms, clock genes, desynchrony, entrainment, human, shift work, sleep

## Abstract

Extending a normal 24 hours day by four hours is unexpectedly highly disruptive to daily rhythms in gene expression in the blood. Using a paradigm in which human subjects were exposed to a 28 hours day, Archer and colleagues show how this sleep-altering forced desynchrony protocol caused complex disruption to daily rhythms in distinct groups of genes. Such perturbations in the temporal organisation of the blood transcriptome arise quickly, and point to the fragile nature of coordinated genomic activity. Chronic disruption of the daily and circadian rhythms in sleep compromise health and well-being and this study reveals potential new molecular targets to combat the disruptive effects of shift work and jetlag.

## Introduction

Over the past 60 years, the study of intrinsic daily or circadian rhythms has established that biological timekeeping mechanisms pervade all aspects of physiology and behaviour. From the 1950s onwards, research on bacteria, plants, flies and rodents laid the foundations for understanding the formal properties of circadian rhythms in behaviour and physiology and in particular, how they are reset by external time cues called Zeitgebers. Such pioneering studies demonstrated that in many animals, including humans, a range of stimuli including environmental light, food availability and physical exercise function as Zeitgebers, synchronising circadian rhythms in behaviour and physiology to the external world [1–3]. Through experimental brain lesion studies, researchers in the 1970s determined that the suprachiasmatic nuclei of the hypothalamus (SCN) contained the circadian clock critical for the expression of circadian rhythms in behaviour and hormones [4, 5]. Subsequently, anatomical and functional studies established how the SCN integrates Zeitgeber and circadian information and conveys this temporal signal to regulate brain and physiology including the daily organisation of the sleep-wake cycle and the release of the pineal hormone, melatonin [1].

At one time, it was believed that the SCN was the only circadian clock in mammals. However, in the late 1990s, the identification and characterisation of the so-called ‘core clock genes and proteins’ that constitute the molecular basis of the SCN circadian clock [6] led to the perhaps startlingly realisation that most, if not all, cells in the mammalian brain and body contain the molecular circadian clockworks [7]. This includes cells in peripheral tissues such as liver, lungs and heart, as well as the blood. The hierarchical model of the circadian system that has emerged is that of the central SCN circadian clock integrating time cue information and conveying neural and blood-borne signals to orchestrate the circadian oscillators in extra-SCN brain sites as well as in the periphery [8–11].

Subsequent molecular profiling of transcript expression in different mammalian tissues sampled throughout the circadian cycle demonstrated that, depending on the tissue type, up to ∼10% of the genome shows circadian variation [12, 13]. This also facilitated the distinction of core clock genes from clock-controlled genes (CCGs). Since the core clock genes are present in many cells, and because CCGs can function as clock-controlled outputs, it is now recognised that both central and ‘local’ clocks can regulate key cellular activities and influence physiological processes such as metabolism and tissue repair (e.g. [14]). Indeed, fully functional local clocks are necessary for appropriate circadian control of tissue specific physiology in the periphery. For example, a recent investigation, which selectively rescued circadian clock mechanisms in the brain revealed that while behavioural rhythms were restored, circadian output from the liver remained abnormal [10]. Further, knowledge of the molecular clock and CCGs allows determination of the molecular actions of Zeitgebers and other conditions known to perturb the circadian system. For example, in experimental mice, brief exposure to light during the night rapidly increases the expression of the core clock genes *Per1-2* and their protein products [15]. Tracking of core clock genes in peripheral tissues indicates that local molecular clocks lag that of the SCN by some hours [16]. This temporal arrangement can be overridden, however, by periodic administration of relevant signals such as food availability, which reset peripheral oscillators, and hence some tissue-specific CCGs, to influence local rhythmic physiology [17].

Appropriate synchronisation of the circadian axis, and by extension, all body systems, is important for good health and personal well-being [18]. Misalignment of the circadian system, which can arise through chronic exposure to shift work schedules, elevates the risk of developing certain cancers, as well as cardiovascular and metabolic disorders [19, 20]. One key circadian rhythm in brain state that is overtly disrupted by shift work is the sleep-wake cycle. Sleep, itself a critical physiological process [21, 22], is under both circadian and homeostatic control; the SCN acts to signal sleep onset, while homeostatic influences increase with time awake to elevate the desire for sleep, or sleep pressure [23]. Disruption and deprivation of sleep is detrimental to cognitive performance and mental health [24], and leads to increased body weight and associated negative health consequences [25, 26]. Further, sleep loss can also feedback to alter the circadian rhythm in melatonin [27], demonstrating a bidirectional relationship between sleep and the circadian system. Indeed, a recent investigation of the consequences of insufficient sleep on rhythmicity in the human peripheral transcriptome identified a marked reduction in rhythmic transcripts following insufficient sleep [28]. Understanding the molecular changes that occur during sleep disruption therefore requires experimental designs that distinguish circadian and homeostatic influences, and this has been addressed in a recent investigation by Archer et al. [29]. This research assesses rhythmicity in the human blood transcriptome in human subjects whose sleep/activity schedule and endogenous circadian rhythms have been experimentally dissociated.

## A forced desynchrony protocol dissociates endogenous circadian rhythms from sleep

**Figure 1:**
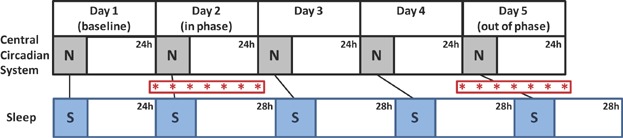
Schematic time series diagram showing experimental design. After a 24 hours baseline (Day 1), 28 hours cycles of sleep-wake, activity, meals and dim light:dark were imposed upon subjects (lower section; blue boxes indicate times of sleep/inactivity imposed by the experimental schedule). The central circadian system is unable to synchronise with this schedule and ‘free-runs’ with a period of near to 24 hours (upper section; grey boxes indicate times of biological night governed by the circadian system). A series of seven blood samples were taken on Day 2, when the central circadian system and sleep schedule were in phase, and again on Day 5, when the circadian clock and sleep schedule were out of phase. Red asterisks show times of blood sample collection. To confirm the timing of biological night, melatonin levels, which are high during biological night, were analysed from Day 2 and 5 blood samples and times of sleep were measured using polysomnography. The effects of scheduling sleep and associated rhythms in and out of phase with biological time on rhythmic expression of the human blood transcriptome were determined using microarray analysis on RNA transcripts extracted from the collected blood samples. N, biological night; S, scheduled sleep.

In this study, Archer et al. [29] use a forced desynchrony protocol to dissociate the endogenous circadian system, reported by rhythms in the blood concentration of the hormone melatonin, from a set of behavioural and physiological rhythms including sleep-wake, feeding, metabolism and locomotor activity. Forced desynchrony is an established protocol for human sleep studies that schedules participants sleep-wake cycles, feeding and light exposure to a 28 hours day [30]. Performed under very dim lighting of less than 5 lux during light phases, the extreme length of this schedule falls outside the limits of entrainment of the intrinsic circadian system which, unable to entrain, oscillates at its endogenous period of close to 24 hours. Under baseline 24 hours day conditions the melatonin rhythm oscillates in phase with sleep. However, with these endogenous oscillations maintained at ∼24 hours and an externally imposed 28 hours schedule of sleep and associated rhythms, this paradigm results, on the 4th day, in the onset of melatonin expression and sleep occurring 12 hours apart. The authors assessed the effects of this dissociation between sleep and biological time on rhythmic expression of the human peripheral transcriptome. Blood samples were collected at seven time-points, four hours apart, on the 1st day of the 28 hours schedule, when sleep and melatonin were still aligned and again on the 4th day when these oscillations were in antiphase (see Fig. 1). Archer and colleagues then performed microarray analysis on these samples, on an unbiased transcriptome-wide basis. These analyses were used to determine the baseline rhythmicity of human blood transcripts when sleep and biological time were aligned, and to assess alterations to the rhythmicity, temporal profiles and absolute levels of these transcripts when rhythms were dissociated. The authors then further characterised their data through the use of gene ontology (GO) analysis to cluster genes of interest into functional groupings based on both biological processes, such as transcription, translation and protein localisation and molecular functions, such as RNA binding and methyl transferase activity. The authors finally assessed potential functional interactions between transcripts that showed altered expression under forced desynchrony using direct network interaction analysis to identify a number of promiscuous transcription factors altered under forced desynchrony that may mediate its wider effects on the transcriptome at large.

## Dissociating endogenous circadian rhythms from sleep disrupts circadian expression of the human transcriptome

These analyses revealed that the transcripts for ∼6% of all genes assessed were expressed with a significant circadian rhythm under baseline conditions, a figure broadly consistent with previous assessments of rhythmicity in the mammalian peripheral transcriptome [28, 31, 32]. Rhythmic transcripts represented a wide range of biological processes and clustered into two temporally distinct groups, ‘day-peaking’ and ‘night-peaking’ transcripts, with functional processes differing between these groups. The biological processes represented by transcripts clustered in the day- and night-peaking groups offered a pleasingly intuitive breakdown of functions: day-peaking transcripts were generally associated with responses to the stresses and insults of the daily active phase (relating to immunity and infection: e.g. wound, defence and stress responses) and night-peaking transcripts were generally associated with restorative processes consistent with recovery during the daily inactive phase, mostly relating to gene expression, protein production and targeting.

**Figure 2:**
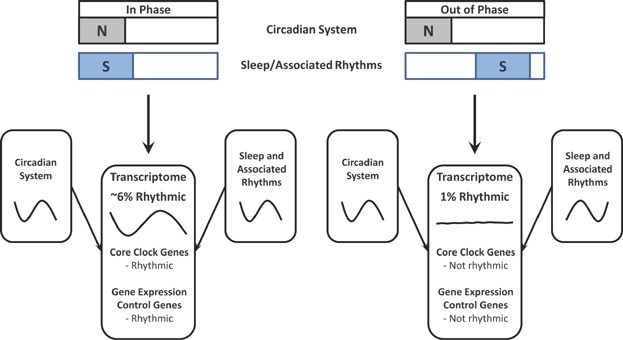
Circadian rhythmicity in the human blood transcriptome is rapidly and profoundly disrupted when endogenous circadian processes are mistimed relative to an experimentally imposed schedule of sleep-wake and associated rhythms. Rhythmic gene expression can be driven by either one, or both, of these influences and when they are poorly aligned overall rhythmicity is reduced. Plotting conventions as for Fig. 1 (blue boxes, S, indicate times of sleep/inactivity; grey boxes, N, indicate times of biological night).

Remarkably, dissociating the timing of sleep and associated locomotor, feeding and metabolic rhythms from endogenous circadian processes had a rapid and profound disruptive effect on the temporal organisation of the transcriptome, such that when sleep and melatonin were in antiphase, the percentage of rhythmic transcripts was reduced to only 1% (see Fig. 2). Further, transcripts for almost all genes that were rhythmic at baseline failed to oscillate at the antiphase stage of forced desynchrony. This marked disruption of normal rhythmicity included the majority of the canonical core clock genes (e.g. *BMAL1*, *PER2* and *CRY2*), and affected both day- and night-peaking clusters, though transcripts for genes associated with functions predominant in the night-peaking cluster, such as protein targeting and translation, were prominently represented. The extensive nature of this disruption to rhythmicity in the peripheral transcriptome is further emphasised upon considering that those strongly rhythmic transcripts not altered by forced desynchrony are perhaps not reflective of the peripheral transcriptome at large. Indeed, such transcripts were generally associated with tissue-specific processes relevant to circulation, such as blood cell development.

To nuance their analysis of this data set further, Archer et al. next investigated transcripts that did not lose rhythmicity altogether, but did show alteration of their temporal expression profiles between the in and out of phase conditions. In this analysis, an enormous 34% of transcripts were altered by forced desynchrony, and included transcripts for genes related to chromatin modification, ribosomal function, transcription and translation; hence defining a clear effect of mistimed sleep (and associated rhythmic functions) on the control of these critical molecular processes. The authors used a mathematical model to determine whether transcripts were most responsive to endogenous circadian time, sleep/external time or a combination of both, and were able to identify groups of transcripts that could be classified in each of these categories. This further illustrates the disorganised nature of transcriptome rhythmicity under forced desynchrony. Consistent with this, the authors also identified an overall down-regulation in transcripts for a large number of genes involved with molecular metabolism and DNA/RNA binding, whilst the small number of transcripts up-regulated by forced desynchrony were generally associated with protective processes such as peroxiredoxin and oxygen transporter activity. With this combined data set, Archer and colleagues present an acute disruption of peripheral circadian function and normal transcriptional/translational processes by the dissociation of sleep and associated rhythms from central circadian function. This observation is strongly suggestive that the observed changes are detrimental to normal physiology.

## Promiscuous regulators of gene expression likely promulgate the effects of forced desynchrony through the human peripheral transcriptome

To address the widespread nature of disrupted rhythmicity of the human transcriptome by forced desynchrony of melatonin and sleep (and by extension, rhythms in feeding/metabolism/locomotion), the authors mapped possible functional interactions between transcripts with altered temporal expression under forced desynchrony. Using this direct network interaction analysis, transcripts were identified that code for a number of proteins that promiscuously regulate transcription, including SP1, EP300 and CREB binding protein. Together these proteins have the potential to regulate the expression of a large number of downstream genes and – coupled with disruption of the canonical core clock genes and the knowledge that promoters of multiple clock genes contain SP1 binding sites – this offers a mechanism for the extensive disruption of the temporal order of the human peripheral transcriptome observed in this paradigm. Further mechanisms likely to contribute to the proliferation of disrupted circadian expression through the peripheral transcriptome under forced desynchrony include histone acetylation and methylation. Indeed, transcripts of genes involved in both of these processes (e.g. EP300 and CREB binding protein, as well as HAT1 and the MLL family of genes) were identified by Archer and colleagues as altered, and both histone acetylation and methylation have recently been shown to contribute to widespread circadian control of gene expression [33, 34]. Moreover, Azzi et al. [35] recently demonstrated a key role of dynamic DNA methylation in the central control of circadian function, raising the intriguing possibility that this process acts as a novel mechanism for the circadian control of gene expression in the periphery. The SCN circadian oscillator provides the principal drive for the timing and rhythmicity of circulating melatonin rhythms, which remain unaltered under forced desynchrony, indicating that the core SCN clockwork is not disrupted. The extreme disruption of the peripheral transcriptome observed here, therefore, demonstrates that feedback from those rhythms that are dissociated from the central clock, including sleep, feeding and metabolism and locomotor rhythms, provides critical timing information to the peripheral transcriptome.

A caveat to the authors' interpretation of their findings is that considerable emphasis is placed on rhythms in sleep-wake behaviour having been dissociated from melatonin, as is conventional when employing the forced desynchrony protocol used here [36]. However, a potential confounding aspect of this assumption is that no evidence is offered to demonstrate that sleep-wake cycles are indeed the critical function of relevance. As the authors themselves acknowledge, cycles of darkness and light, as well as feeding and fasting with accompanying metabolic oscillations, are associated with cycles of sleep-wake; in the experimental paradigm used here feeding rhythms are scheduled to a 28 hours period in conjunction with sleep and, while not commented on directly, a 28 hours locomotor activity and arousal rhythm is also imposed by the cyclic 28 hours scheduling of sleep and wake. As such, feeding and activity/arousal rhythms (as well as the dim light:dark cycle) also dissociate from the melatonin rhythm and remain in phase with sleep. These stimuli and activities could themselves be critical, in addition to sleep, for producing the effects observed here. As the current experiments are performed under very dim light:dark cycles, using lights of <5 lux during lights-on, a robust effect of external lighting is unlikely to be a major confounding factor, though it cannot be completely ruled out.

## Conclusions

In summary, the study by Archer and colleagues elegantly demonstrates significant and unexpectedly large disruptions in the circadian transcriptome of the blood that arise quickly following desynchrony of the circadian axis from the external world. This demonstrates the importance of appropriate coordination of internal temporal programmes with environmental Zeitgebers, and is highlighted by the more profound molecular disruptions arising from mistimed sleep [29] than were seen in a similar study by this group on sleep insufficiency [28]. Notably, a recent investigation of the effects of sleep on academic achievement revealed sleep timing as having a stronger correlation with performance than sleep duration or quality [37]. Beyond the transcriptome, other circadian-regulated molecular changes are also possible. A recent study of the liver proteome indicated that daily changes in peak expression of some proteins do not require rhythmic changes in mRNA expression [38, 39]. This implies that diurnal control of translational and post-translational processes contributes to local clock physiology. If such mechanisms are widespread in peripheral circadian oscillators, then conceivably translational/post-translational processes in the blood may also be subject to disruption by forced desynchrony. Follow-up studies targeting the blood proteome are necessary to investigate this possibility. Collectively, this study identifies molecular signalling pathways, the activities of which could potentially be targeted for combating the effects of jetlag or exposure to severe shift-work schedules.

## Abbreviations

CCG: clock-controlled gene
SCN: suprachiasmatic nuclei
